# An automated system for quantitative analysis of Drosophila larval locomotion

**DOI:** 10.1186/s12861-015-0062-0

**Published:** 2015-02-24

**Authors:** Boanerges Aleman-Meza, Sang-Kyu Jung, Weiwei Zhong

**Affiliations:** Department of BioSciences, Rice University, Houston, TX USA

**Keywords:** Locomotion, *Drosophila melanogaster*, Video-tracking, Automatic phenotyping

## Abstract

**Background:**

*Drosophila* larvae have been used as a model to study to genetic and cellular circuitries modulating behaviors. One of the challenges in behavioral study is the quantification of complex phenotypes such as locomotive behaviors. Experimental capability can be greatly enhanced by an automatic single-animal tracker that records an animal at a high resolution for an extended period, and analyzes multiple behavioral parameters.

**Results:**

Here we present MaggotTracker, a single-animal tracking system for *Drosophila* larval locomotion analysis. This system controls the motorized microscope stage while taking a video, so that the animal remains in the viewing center. It then reduces the animal to 13 evenly distributed points along the midline, and computes over 20 parameters evaluating the shape, peristalsis movement, stamina, and track of the animal.

To demonstrate its utility, we applied MaggotTracker to analyze both wild-type and mutant animals to identify factors affecting locomotive behaviors. Each animal was tracked for four minutes. Our analysis on Canton-S third-instar larvae revealed that the distance an animal travelled was correlated to its striding speed rather than the percentage of time the animal spent striding, and that the striding speed was correlated to both the distance and the duration of one stride. Sexual dimorphism was observed in body length but not in locomotive parameters such as speed. Locomotive parameters were affected by animal developmental stage and the crawling surface. No significant changes in movement speed were detected in mutants of circadian genes such as *period* (*per*), *timeout*, and *timeless* (*tim*). The MaggotTracker analysis showed that *ether a go-go* (*eag*), *Shaker* (*Sh*), *slowpoke* (*slo*), and *dunce* (*dnc*) mutant larvae had severe phenotypes in multiple locomotive parameters such as stride distance and speed, consistent with their function in neuromuscular junctions. Further, the phenotypic patterns of the K^+^ channel genes *eag*, *Sh* and *slo* are highly similar.

**Conclusions:**

These results showed that MaggotTracker is an efficient tool for automatic phenotyping. The MaggotTracker software as well as the data presented here can be downloaded from our open-access site www.WormLoco.org/Mag.

**Electronic supplementary material:**

The online version of this article (doi:10.1186/s12861-015-0062-0) contains supplementary material, which is available to authorized users.

## Background

The fruit fly *Drosophila melanogaster* is a highly popular model organism for behavioral studies. *Drosophila* larvae display a rich collection of locomotive behaviors such as peristaltic crawling, pausing, and turning [1,2]. Automatic, quantitative analysis of these behaviors is often needed to investigate the molecular and cellular circuitry modulating such behaviors.

There are two types of systems providing such analysis: single-animal tracker and multi-animal tracker. A multi-animal tracker records from a fixed position and analyzes all animals present in the view. A single-animal tracker often changes its position to follow one animal. In general, multi-animal trackers provide a higher throughput, and single-animal trackers enable a higher resolution. As single-animal trackers can use a higher magnification, more phenotypic details can be extracted from the images and videos.

An open-access multi-animal tracker with many powerful features has been successfully designed and used for *Drosophila* larval locomotion analysis [3,4]. In contrast, there is no feature-rich single-animal tracker for *Drosophila* larval locomotion analysis. The most used DIAS system was originally developed to study the crawling of amoeboid cells [5-8]. While it analyzes features such as speed and turns, it lacks *Drosophila* specific parameters such as stride frequency. The software is also not open access. Alternative software such as customized ImageJ plug-ins and Matlab scripts have been used to measure several additional *Drosophila* larval locomotion parameters [9,10]. However, these software programs are often limited to measurements of few parameters and specific experimental designs.

Here we present MaggotTracker, an automatic single-animal tracking system for *Drosophila* larval locomotion analysis. MaggotTracker has several advantages over existing single-animal systems. 1) It analyzes more *Drosophila* larval locomotive parameters. The system measures over 20 parameters, ranging from the duration and distance of one stride to the shape of the entire track. 2) It provides higher resolutions, allowing detailed parameter measurements. For example, speed is measured at 13 equal-distance positions along the body. 3) It resolves the conflict of recording time and resolution by moving the microscope stage to keep the animal in the view field. While most current studies are limited to about 30 seconds of high-resolution recording [6,10], the MaggotTracker has no limitations on how long an animal can be tracked and recorded even at the highest resolution. In this study, we demonstrate a 4-minute recording for each animal. 4) It is free to use. Written in Java, it does not require any commercial software such as LabView or Matlab. 5) It is open source, enabling future development to accommodate new analysis needs. The source codes can be downloaded at WormLoco.org/Mag.

## Results

### Hardware components of MaggotTracker

**Figure 1:**
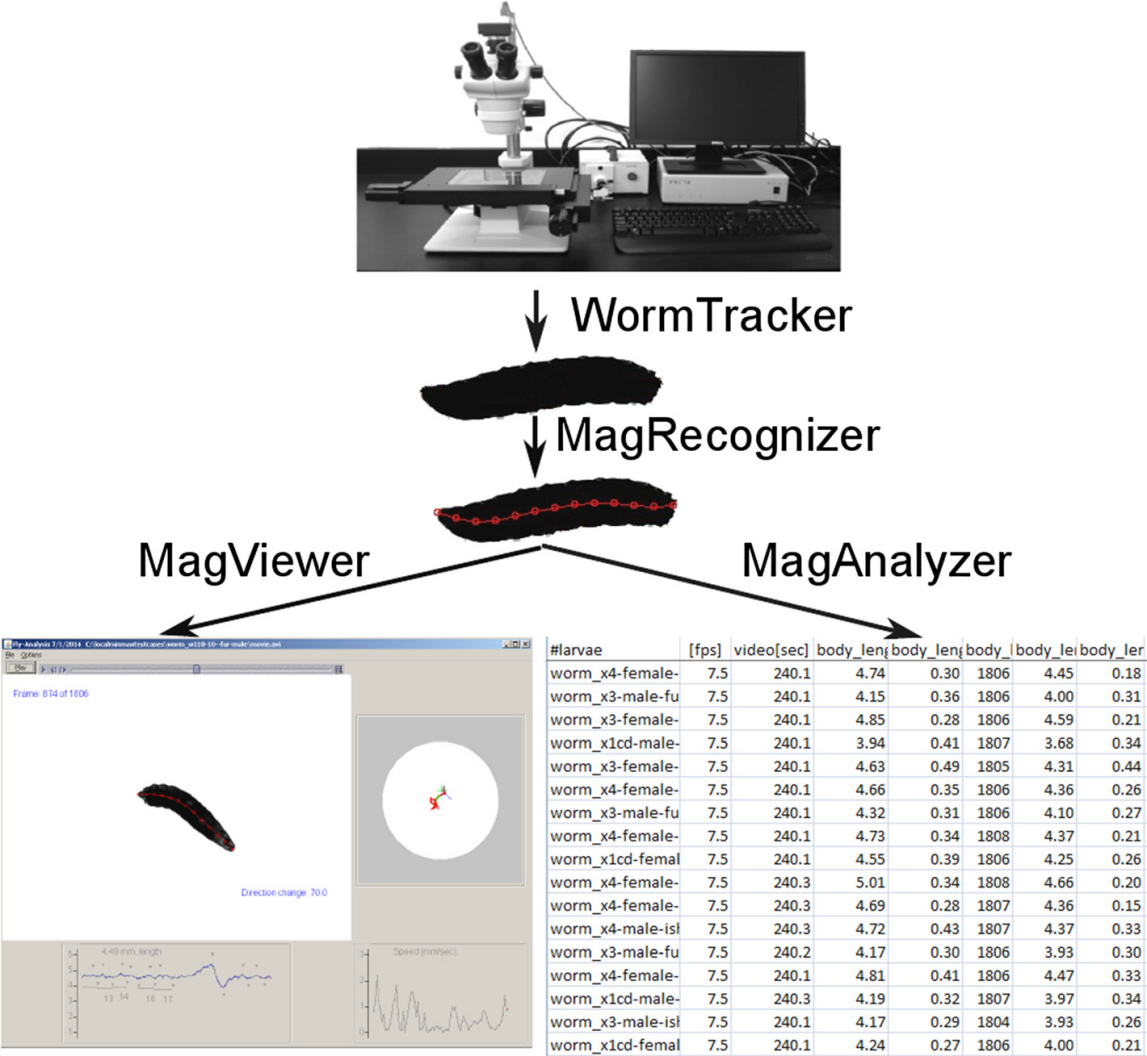
**Components of the MaggotTracker system.** WormTracker controls the hardware to track one animal and record a video. MagRecognizer extracts 13 points equally distributed along the midline of the animal. MagViewer displays the values of animal length and speed over time while simultaneously playing the video and showing the track of the animal. MagAnalyzer conducts batch processing of videos to extract mean parameter values over the entire recording time for all videos.

The hardware for MaggotTracker is composed of a digital camera, a dissecting microscope, a motorized stage, and a computer that controls the camera and the stage (Figure 1). We used a high magnification (50×) microscope as we initially built this system for phenotyping of the much smaller animal *C. elegans* [11]. A setup with a lower magnification microscope or even no microscope should be sufficient for *Drosophila* larval tracking.

The system was placed in a 20°C environmental room so that all experiments were conducted under the same temperature. During tracking, an animal was placed on a 10 cm Petri dish filled with 1.5% agar. A transparent, 1.5-cm-wide plastic ring was placed on the outer rim of the agar to prevent the animal from crawling to the edge of the plate (Figure 1). To optimize image processing, the camera was set to a high contrast so that the animal appeared black and the background was white.

### Software components of MaggotTracker

The software for MaggotTracker has four components, WormTracker, MagRecognizer, MagViewer and MagAnalyzer (Figure 1). All programs were written in Java. WormTracker and MagViewer work on PC computers while the other two programs work on both PC and Mac computers.

WormTracker records a video and moves the motorized stage so that the animal remains in the viewing center. In addition to a video, the program generates a text file listing changes of the stage coordinates over time. This is the only component in the software that is not developed by us because several such programs already exist for monitoring *C. elegans* locomotion [12-14]. Any of these programs would suffice. We used the WormTracker program developed by the Schafer lab [15].

MagRecognizer reduces the animal to 13 evenly distributed points along the midline of the animal. It also uses the record of stage coordinates generated by the WormTracker to compute the position of the animal on the plate. It outputs the result in a text file listing the coordinates of the 13 points for each image frame in the video. This file serves as the input for both MagViewer and MagAnalyzer.

MagViewer dynamically displays the instantaneous values of locomotive parameters as the user plays a video. It generates a text file detailing the locomotive parameter values for each time point. It also generates an image file tracing the tracks of the animal.

MagAnalyzer performs batch processing of all videos in a given folder. For each video, it computes the averages of parameter values over the entire duration of the video. This program outputs a text file listing all videos and their parameter values, which can be directly imported into a database.

All software source codes can be freely downloaded [16]. The website also provides open-access documents such as a user manual, an installation instruction, and detailed experimental protocols.

### MaggotTracker measures multiple locomotive parameters

**Figure 2:**
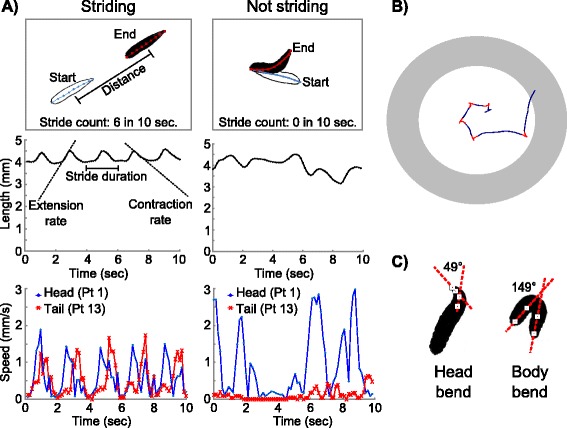
**Sample parameters measured by MaggotTracker.** More parameters measured by MaggotTracker can be found in Table 1. **A)** MaggotTracker measures animal movements. Two 10-second videos of the same animal showed the differences between striding and non-striding movements. Several parameters are measured during the striding phase only. For example, stride duration is the time for one peristalsis cycle; extension and contraction rates measure the rate of length changes. Other parameters such as length and speed are measured at all time. Some parameters such as speed are measured for each point along the midline from head to tail. **B)** MaggotTracker traces animal tracks. Grey ring shows the position of the plastic ring used to prevent animals from crawling off the agar. Direction change points are marked red. **C)** MaggotTracker measures animal shape. Head angle and body angle are calculated to determine whether there is a head bend or a body bend.

*Drosophila* larval locomotion patterns may be categorized as striding or non-striding [17] (Figure 2). When an animal is striding, it displays a peristaltic movement: the animal extends and contracts rhythmically; it moves linearly covering a significant distance; the head and tail of the animal move at a similar speed during the extending and contracting phase, respectively (Figure 2A). In contrast, a non-striding animal shows no rhythm in body length changes; it turns its head sideways and bends its body without traveling much distance; such head movements also cause the instantaneous speed of the head to be much greater than that of the tail (Figure 2A).

Backward movement was rarely observed in our tracking system. Over 99% of all the animals we analyzed showed no backward movement at all. For the few animals that did move backward, the backward movement lasted only one or two strides during the four-minute video. Therefore, the MaggotTracker does not specifically analyze backward movements. A previous study showed a much higher percentage of backward movement [9]. The difference may be caused by different experimental conditions (linear crawling in a channel in the other study vs. free crawling on an agar plate in our assay).

MaggotTracker measures over 20 parameters to capture both of these locomotive patterns (Table 1). These parameters describe the shape, peristaltic movement, stamina, and track of the animal. Some of these parameters such as speed are measured for each of the 13 points along the body. In addition, some parameters such as length are measured for instantaneous values at each time point using MagViewer, as well as average values over the entire video using MagAnalyzer.

**Table 1:** **Parameters measured by MaggotTracker**

**Parameter**	**Unit**	**Definition of Parameter**
Animal shape		
Body Length	mm	The length of the midline of the larva measured using all frames.
Body Length Contracted	mm	Body length measured using a subset of frames when the larval length reaches the local minimum during a stride. ^1^
Body Length Extended	mm	Body length measured using a subset of frames where the larval length reaches the local maximum during a stride. ^1^
Time Head Bending	%	Percentage of time when the larva has a head angle of over 45 degrees. ^2, 3^
Time Body Bending	%	Percentage of time when the larva has a body angle of over 45 degrees. ^2, 3^
Time Bending	%	Percentage of time when the larva has either its head or body angle over 45 degrees. ^2, 3^
Peristalsis movement		
Speed	mm/sec	The positional change of the center point over time. ^2, 4^
Time Striding	%	Percentage of time when the larva is striding. ^2, 3^
Speed Striding	mm/sec	Speed measured using a subset of frames when the animal is striding. ^1, 2, 4^
Stride Duration	sec	Time duration of one stride. ^1, 2^
Stride Distance	mm	Distance traveled by the center point during one stride. ^1, 2, 4^
Contraction Rate	mm/sec	The rate of body length change during the contraction phase of a stride. ^1, 2^
Extension Rate	mm/sec	The rate of body length change during the extension phase of a stride. ^1, 2^
Stamina		
Stride Count	counts/min	Total number of strides over the video length. ^3^
Run Distance	mm	Average distance traveled in a run. A run is defined as a period when the animal is striding continuously. ^3^
Run Duration	sec	Average time duration of a run. ^3^
Run Stride Count	counts/min	Average number of strides of a run. ^3^
Run Count	counts/min	Total number of runs over the video length. ^3^
Track		
Distance	mm/min	Total distance traveled by the center point over the video length. ^3^
Direction Change	%	Percentage points on the track where the animal is changing its direction. ^3^
Time Inside	%	Percentage of time the animal is away from the plastic ring. ^3^

^1^ These parameters are only measured for the video segments when the animal is striding.

^2^ These parameters are measured in three ways: overall, inside, and outside. Overall values evaluate the whole video. Inside values evaluate the animal when it is away from the plastic. Outside values evaluate the animal when it is close to or on the plastic ring. Overall values were used by default unless otherwise specified.

^3^ These parameters are measured over the entire video. They have one value data point from each video. The other parameters are measured for each frame. The mean value was used for each video.

^4^ These parameters are measured for each of the 13 points along the midline. Values for the center point, Point 7, were used by default unless otherwise specified.

### Variations of locomotive parameters

As the first application of the MaggotTracker, we used it to examine the locomotive behaviors of 623 wild-type Canton-S animals. We measured the coefficient of variation (CV), i.e., the ratio of the standard deviation divided by the mean, for each parameter. Most parameters showed a low variation with CV values of less than 50% (Table 2). In particular, all parameters measuring the peristalsis movement showed low CV values of less than 35% (Table 2). These data suggested that these parameters are good indicators for phenotypic assessment. For the rest of the paper, we focus on these less variable parameters unless otherwise specified.

**Table 2:** **Coefficient of variation (CV) of parameters**

**Parameter**	**Animal-to-animal variation**		**Day-to-day variation**	
	**n = 623**		**n = 54**	
	**Mean ± S.D.**	**CV (%)**	**Mean ± S.D.**	**CV (%)**
Animal shape				
Body Length	4.34 ± 0.35	**8.2**	4.33 ± 0.26	**6.1**
Body Length Contracted	4.12 ± 0.34	**8.3**	4.12 ± 0.26	**6.3**
Body Length Extended	4.63 ± 0.38	**8.2**	4.62 ± 0.27	**5.9**
Time Head Bending	0.01 ± 0.01	107.3	0.01 ± 0	**37.9**
Time Body Bending	0.1 ± 0.07	67.8	0.1 ± 0.03	**25.6**
Time Bending	0.11 ± 0.07	65.9	0.11 ± 0.03	**24.6**
Peristalsis movement				
Speed	0.62 ± 0.2	**32.9**	0.62 ± 0.15	**24.7**
Time Striding	0.76 ± 0.13	**16.7**	0.76 ± 0.05	**6.7**
Speed Striding	0.62 ± 0.19	**30.4**	0.62 ± 0.14	**22.7**
Stride Duration	1.61 ± 0.38	**23.4**	1.61 ± 0.24	**14.9**
Stride Distance	0.89 ± 0.16	**18.4**	0.89 ± 0.12	**13.0**
Contraction Rate	0.69 ± 0.14	**20.9**	0.69 ± 0.08	**12.2**
Extension Rate	0.82 ± 0.19	**22.4**	0.82 ± 0.11	**13.4**
Stamina				
Stride Count	29.91 ± 8.46	**28.3**	29.89 ± 5.12	**17.1**
Run Distance	13.92 ± 8.95	64.3	13.88 ± 4.66	**33.6**
Run Duration	23.53 ± 14.78	62.8	23.48 ± 7.06	**30.1**
Run Stride Count	14.8 ± 8.83	59.6	14.77 ± 4.17	**28.2**
Run Count	2.25 ± 0.93	**41.4**	2.25 ± 0.44	**19.4**
Track				
Distance	33.55 ± 10.77	**32.1**	33.49 ± 8.07	**24.1**
Direction Change	0.25 ± 0.14	56.2	0.25 ± 0.06	**23.5**
Time Inside	0.61 ± 0.24	**39.0**	0.61 ± 0.12	**20.0**

Animal-to-animal variations: each data point is an animal; n is the number of animals, pooled from all experimental dates. Day-to-day variation: each data point is the mean value from animals tracked on the same day; n is the number of dates. S.D., standard deviation. **Bold text**, coefficient of variation (CV) <50%.

Several parameters showed high variation with CV values higher than 50%. These parameters include bending time for head and/or body, number of direction change points on the track, stamina parameters measuring the distance, duration, and stride count for each run during which the animal strides continuously.

### Correlations of locomotive parameters

**Figure 3:**
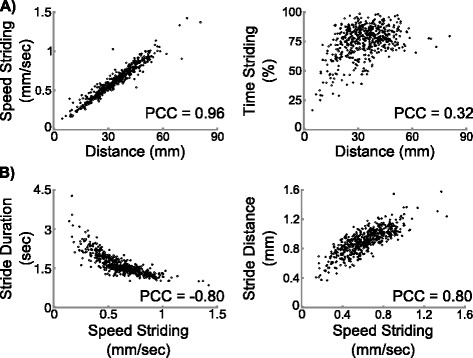
**Correlation of parameters. A)** Scatter plots showing that distance travelled is more correlated with speed striding than time striding. **B)** Scatter plots showing that speed striding is negatively correlated to the duration of one stride and positively correlated to the distance of one stride. PCC, Pearson correlation coefficient.

As Canton-S animals showed a range of distanced travelled during the 4 minute time, we asked whether an animal covered more distance by spending more time striding or by moving faster when striding. Correlation analysis of the parameters showed that distance is highly correlated to striding speed, but not striding time for these animals (Figure 3A), suggesting that an animal covers more ground by striding faster rather than spending more time striding.

We next asked whether a faster animal had bigger or faster steps. We analyzed the correlation between the striding speed and the distance and duration of one stride (i.e., one step). Striding speed positively correlated with stride distance and negatively correlated with stride duration (Figure 3B), suggesting that a faster animal moved with both bigger and faster steps. These results are consistent with a previous report by Berrigan and Pepin [2]. Another report by Heckscher et al. showed that speed has a stronger correlation with stride duration than distance [9]. The difference might be caused by experimental conditions: Heckscher et al. used a channel so that the movement of the animals were restricted to a linear fashion, while the animals were crawling on the surface of an agar plate, a two-dimensional space, in our experiment and the assays of Berrigan and Pepin.

Several other parameters also showed high correlations with the absolute values of Pearson correlation efficient above 0.7. Stride count is positively correlated to striding time and speed, while negatively correlated to the duration of one stride (Additional file 1: Table S1). Body length measurements, such as average length, contracted body length, and extended body length, are highly correlated among themselves (Additional file 1: Table S1).

### Effects of gender, development, and medium surface on locomotion

**Figure 4:**
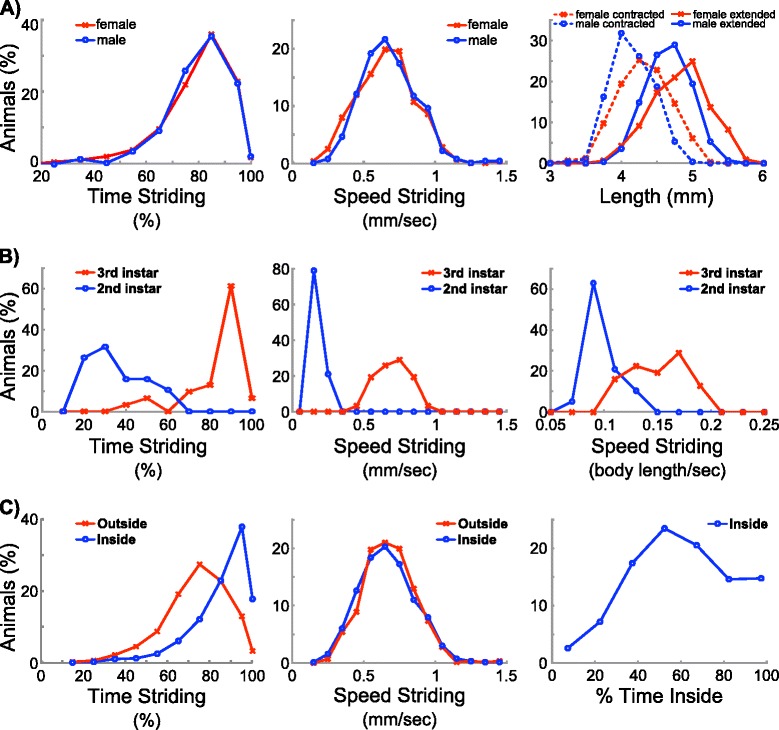
**Factors affecting locomotive behaviours.** All graphs are histograms comparing locomotive parameter values such as time striding (left) and speed striding (middle) of males vs. females **(A)**, 2nd vs. 3rd instar animals **(B)**, and animals traveling inside on agar vs. outside on the plastic ring **(C)**. Graphs on the right show sexual dimorphism in body length **(A)**, body length normalized striding speed **(B)**, and percentage of time that animals spent inside on the agar **(C)**.

While it is known that female *Drosophila* larvae are bigger than males, it is unknown whether there are sexual dimorphisms in locomotive behaviors. To investigate this, we used the MaggotTracker to analyze 329 female and 283 male Canton-S wandering third-instar larvae. These animals came from the same set of the 623 animals used in previous parameter correlation and variation analysis. Our data showed that females have significantly longer body length than males (Figure 4A), consistent with previous reports [18]. However, despite of the size difference, males and females showed no significant differences in all other locomotive parameters we measured (Figure 4A, Additional file 2: Table S2). For example, they are indistinguishable in striding speed and striding time (Figure 4A).

We also compared second and third instar larvae, and found that they had drastic difference in most locomotive parameters (Figure 4B, Additional file 2: Table S2). Second instars moved at a slower speed and spent less time striding (Figure 4B). To examine whether the slower speed of second instars are due to its smaller size, we normalized the speed with body length. The speed difference between second and third instars is smaller but still significant when it is length-normalized (Figure 4B). 89% of second instar larvae moved less than 11% of their body length per second, while 84% of third instar larvae moved over 11% of their body length per second (Figure 4B). These data suggested that smaller body length is not the only contributing factor of the slower speed for second instars. As the contraction and extension rates of second instars are significantly lower (Additional file 2: Table S2), it is likely that second instars are weaker. It is possible that other factors such as lack of motivation may also contribute to the slower speed of second instars.

Because our setup used a plastic ring to prevent the animals from crawling out, we analyzed our data on 623 Canton-S third instars to assess how the locomotion behaviors changed when the animals moved from agar to the plastic ring. No significant difference was observed in most locomotive parameters (Additional file 2: Table S2). For example, the striding speed of the animals remained the same on both surfaces (Figure 4C). One exception was that the animals spent significantly less time striding when they were next to or on the plastic (Figure 4C). As most animals spent the majority of their time away from the plastic on the agar (Figure 4C), the plastic ring in our setup may have little impact on the overall locomotive parameter values.

### Use of MaggotTracker to quantify mutant phenotypes

**Figure 5:**
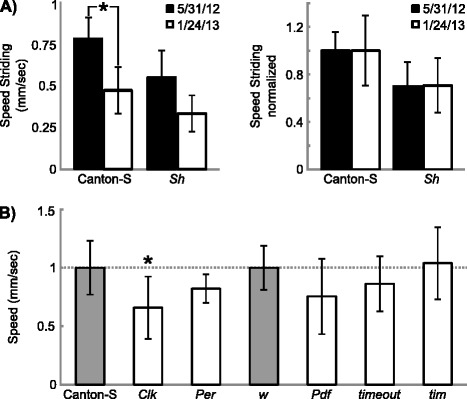
**Use MaggotTracker to examine effects of circadian genes on speed. A)** Left, striding speed of Canton-S and Sh animals measured in two experiments (5/31/12 and 1/24/13). Right, striding speed normalized using same-day-tracked Canton-S animals. n ≥ 9 animals for each genotype in each experiment. *, *p* < 0.001, student’s *t*-test. **B)** Normalized speed values from mutants of multiple circadian genes. Gray bars, control groups. White bars, test mutant groups. n ≥ 11 animals for each genotype. *, *p* < 0.001 between control and mutant groups using one-way ANOVA and Scheffe post hoc test. Bars and error bars are means and standard deviations.

Our primary goal of designing MaggotTracker is to use it for quantitative phenotyping. As locomotive behaviors are affected by many factors such as environmental factors, it is thus important to isolate genetic effects from other factors. To evaluate the influences of non-genetic factors, we examined animals of the same genotype tested on different dates. For example, our Canton-S data were collected on 54 independent trial dates. Overall, little difference in parameter values was observed in most of our experiments conducted on different dates. The variations of mean values from day to day are smaller than the animal-to-animal variations for all parameters (Table 2). However, there are a few cases when parameter values differed significantly for the same genotype tested on different dates (Figure 5A). These cases were all linked to two experimenters and we suspected that the cause of such variations was the small difference in age of the animals or some uncontrolled environmental factors. Normalizing the values using a control strain tested on the same day seemed to eliminate such influences. For example, both Canton-S and *Shaker* (*Sh*) animals showed lower striding speed in the experiment conducted on 2013/01/24 than that of 2012/05/31 (Figure 5A). When the mean value of the same-day-tracked Canton-S animals was used to normalize the values, the normalized *Sh* values are similar in both experiments (Figure 5A). It should be noted that the day-to-day variation in this example represented the most extreme case. The striding speed for Canton-S animals on these two dates (0.47 ± 0.14 and 0.78 ± 0.12) deviated considerably from the mean speed from all dates (0.62 ± 0.19, Table 2) in opposite directions. Since normalization functioned effectively even in such extreme cases, we used normalized values for all our genetic analysis.

The first set of mutants we analyzed was circadian mutants. Locomotor activities in adult flies are regulated by circadian rhythms [19]. While no circadian locomotive rhythms have been reported in larvae, circadian genes are known to regulate the larval light avoidance behavior [19]. As larvae are placed under light in the recording system of MaggotTracker, we questioned whether circadian genes can affect the locomotive patterns under such conditions. We analyzed mutants of various circadian genes such as *Clock* (*Clk*), *period* (*per*), *timeout*, *timeless* (*tim*) and *Pigment-dispersing factor* (*Pdf*). *Pdf, timeout,* and *timeless* mutants were on a *white* (*w*) background, therefore, *w* instead of Canton-S animals were used as the control. For this experiment, the animals were raised under 12-light and 12-hour dark cycles. Third instar larvae for each genotype were analyzed during the light cycle. We first examined the overall speed of the mutants to see whether they have a larval locomotor phenotype similar to that in the adult flies. The overall speed of most mutants showed no significant difference from controls with slightly lower means and comparable variations (Figure 5B, Additional file 3: Table S3). Only *Clk* mutant larvae showed significantly reduced speed (Figure 5B). A similar pattern was observed for other parameters with most mutants showing no significant phenotypes (Additional file 3: Table S3). As *Clk* is a transcription factor upstream of most other circadian genes [19], it may have circadian-independent functions affecting locomotive behaviors. It is also possible that the observed locomotive phenotypes of *Clk* may be caused by a background mutation. A second *Clk* allele or a rescue experiment is needed to confirm the role of *Clk* in regulating locomotive behaviors. Overall, as most circadian mutants showed no phenotypes, our data suggested that circadian mechanisms do not affect the locomotive parameters measured under our experimental conditions.

Next we analyzed a set of mutants that are known to have locomotive phenotypes in adult flies, including mutants of K^+^ channel genes *ether a go-go* (*eag*), *Hyperkinetic* (*Hk*), *Shaker* (*Sh*), *slowpoke* (*slo*), and the Na^+^ channel gene *paralytic* (*para*) [20]. *Sh*, *eag*, and *Hk* mutant adult flies have anesthesia-induced leg shaking [21]. Adult flies of *para* mutants have temperature-induced paralysis [22,23]. We also examined mutants of the c-AMP phosphodiesterase gene *dunce* (*dnc*). While *dnc* mutants were first identified for adult learning defects [24], they also have defects in larval neuromuscular junctions (NMJ) [25]. Although *dnc* mutants appear normal in several general behaviors [24], some adult locomotive phenotypes have been reported with the *dnc*^*1*^ allele such as reduced “centrophobism” (*i.e.*, center avoidance) [26]. Therefore, it is possible that *dnc*^*1*^ animals may also have larval locomotive phenotypes.

**Figure 6:**
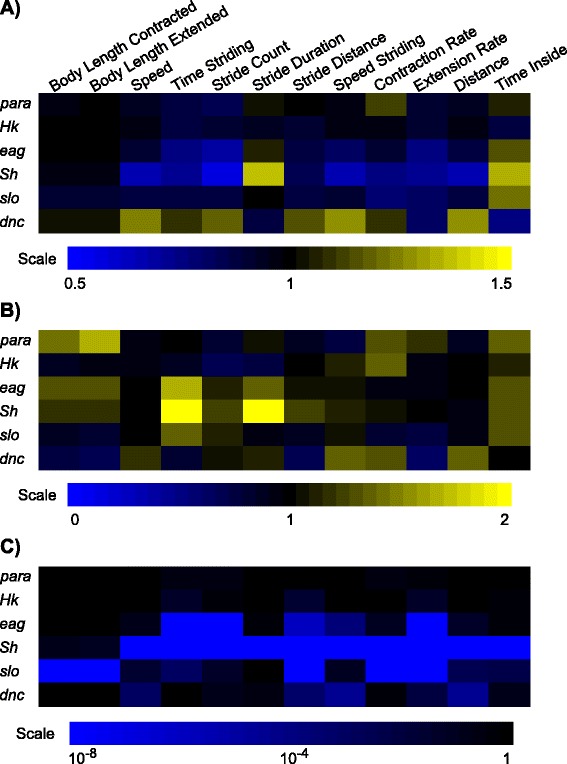
**Use MaggotTracker for phenotypic profiling.** Heat maps showing normalized means **(A)**, normalized standard deviations **(B)**, and *p* values **(C)** for 12 locomotive parameters from six mutants. Normalized means in **(A)** were calculated using mutant means divided by control means. Normalized standard deviations in **(B)** were calculated using mutant standard deviations divided by control standard deviations. In **(A)** and **(B)**, black (normalized value of 1) indicates the same value as control; blue and yellow indicate lower and higher than control values, respectively. In **(C)**, *p* values were computed between the control and the mutant groups using one-way ANOVA and Scheffe post hoc test. *n* ≥ 20 animals for each genotype. Heat map scales were generated using rounded minimum and maximum values and splitting the range into 33 colors.

The MaggotTracker analysis showed that while *para* and *Hk* larvae displayed little locomotive phenotypes, *dnc*, *eag*, and *Sh* larvae had severe phenotypes in multiple locomotive parameters such as stride distance and speed (Figure 6, Additional file 4: Table S4). *Sh* larvae showed the most extensive phenotypes with significant defects (*p* < 0.0001, one-way ANOVA) in ten locomotive parameters (Figure 6C). Some parameters such as time striding were also more variable in *eag* and *Sh* mutants (Figure 6B). However, data from more mutants are needed to detect possible correlations between phenotypic severity and variability of a parameter.

Our phenotypic measurements of *eag*, and *Sh* in stride duration and distance are consistent with previously published results [7]. In both studies, *eag* and *Sh* had no significant phenotypes in stride duration, and showed significant decrease in stride distance. The mean normalized stride distances of *eag* and *Sh* are also similar (*eag*: 0.86 in our study vs. 0.78 in the other; *Sh*: 0.85 in our study vs. 0.80 in the other). One group reported that *para* and *Hk* larvae had slower crawling speed [8] although we did not observe significant differences between these animals and the control Canton-S animals. The discrepancy can be caused by different alleles (*para*^*ts1*^ in our assay vs. *para*^*st76*^ in the other), different experimental conditions such as assay temperature (20°C in our assay vs. room temperature in the other), and media (1.5% agar in our assay vs. 0.7% agarose in the other). It may also be caused by analysis methods because we normalized all data using control animals tracked on the same day.

As MaggotTracker analyzes multiple parameters, the resulting phenotypic profile can provide additional information on phenotypic patterns. For example, mutants of the three K^+^ channel encoding genes *eag*, *Sh* and *slo* showed highly similar phenotypic profiles with a Pearson correlation coefficient of 0.88 between *Sh* and *eag*, 0.71 between *slo* and *eag* (Figure 6A). In contrast, the Pearson correlation coefficient between *dnc* and *eag* is only −0.49 (Figure 6A). These data suggested that Maggotracker is an effective phenotyping tool, detecting differences not only in parameter values but also in phenotypic patterns.

## Discussion

MaggotTracker provides medium-throughput phenotyping. The most labor- and time- intensive part is tracking the animals, as each animal needs to be picked from a vial, sexed under a microscope, and acclimated to the agar plate before being tracked for four minutes. This step takes about 6 minutes per animal. The throughput of this step can be increased by setting up multiple trackers to conduct parallel tracking. Our throughput is 30 videos per hour with one person operating four trackers. The subsequent analysis step takes about 4 minutes per video. This step is fully automatic and can be done in batch processing; therefore, one can simply let the program run overnight to process all videos.

While multi-animal trackers can provide higher throughput of animals examined, single-animal trackers can have unique advantages in analyzing *Drosophila* larval movement in addition to higher resolutions. A recent study showed that *Drosophila* larvae secret pheromones that attract other larvae [27]. Therefore, tracking a single animal can separate locomotive phenotypes from larva-interaction phenotypes such as pheromone-sensing defects.

Additional features can be added to the MaggotTracker in the future to extend its application. For example, the current image settings were not optimized for segment detections. Therefore, the system cannot directly measure coordination defects such as the timing of the contraction of each segment. This could be changed with adjustment of lighting and/or usage of GFP markers [28]. Additional hardware such as LEDs will enable applications such as GFP detection and optogenetic analysis. Our system detected that *sh* and *eag* mutants travelled a shorter distance during a peristalsis cycle, had fewer strides in total, and moved at a slower speed. All these phenotypes could be at least partly contributed by coordination defects [6,29,30]. Therefore, it would be interesting to implement software modules to detect additional phenotypes such as coordination. As our software is open-source, such additional modules can be easily added.

Tools similar to the MaggotTracker have been implemented in other organisms such as *C. elegans* [12-14] for quantitative phenotyping of locomotive behaviors. While most parameters are different as *C. elegans* and *Drosophila* larvae have different crawling patterns (sinusoidal wave vs. peristalsis), parameters such as speed and length are measured in both animals. In this study, we found that *eag* mutants moved at a slower speed and *dnc* mutants moved at a higher speed than Canton-S (Figure 6). Similar phenotypes were found in *C. elegans*, where mutants of the *eag* ortholog *egl-2* also have a reduced speed [31] and mutants of the *dnc* ortholog *pde-4* are hyperactive [32], suggesting some conservation of gene functions. It would be interesting to conduct large-scale quantitative phenotyping of locomotive behaviors in *Drosophila* and compare the results with those from *C. elegans* [31,33] to systematically evaluate such functional conservation. The MaggotTracker provides a useful tool to address such questions.

## Conclusions

We developed MaggotTracker, an automated phenotyping system to analyze *Drosophila* larval locomotion at a high resolution. Analysis of selected wild-type and mutant animals showed that MaggotTracker is an effective tool for quantifying changes in locomotive behaviors.

## Methods

### Animals

The following fly strains were obtained from the Bloomington *Drosophila* Stock Center (IN): Canton-S, *Clk*^*Jrk*^*st*^*1*^, *dnc*^*1*^, *eag*^*1*^, *Hk*^*1*^, *para*^*ST76*^, *per*^*0*^*;ry*^*506*^, *Sh*^*14*^, *st*^*1*^*slo*^*1*^, *y*^*1*^*w;Pdf*^*01*^, *w*^*1118*^*;*PBac{PB} *timeout*^*c06976*^, *w*^*1118*^*;*PBac{WH}*tim*^*f01253*^. *w*^1118^ was kindly provided by Michael Stern.

All stocks were maintained on cornmeal agar following a standard recipe (0.8% yeast, 0.93% soy flour, 6.79% yellow cornmeal, 0.8% agar, 7.1% Karo light corn syrup, and 0.45% propionic acid) by the Bloomington Stock Center [34] at room temperature (22°C).

### Behavioral assay

4**–**5 pairs of animals were transferred to a new vial every two days to obtain a synchronized larvae population. Vials containing larvae were moved to a 20°C environmental room at least 12 hours before tracking so that animals could acclimate to the temperature. Unless otherwise specified, wandering third instar larvae were used. Each larva was taken out of the wall of the vial using bristles of a paint brush, and placed onto a 10 cm Petri dish plate containing 1.5% agar. A transparent, 1.5-cm-wide plastic ring was placed on the outer rim of the agar to prevent the animal from crawling to the edge of the plate. The animal was observed under a dissecting microscope to determine its sex, and ensure that it had stopped eating. The animal was then left on the plate for at least one minute to acclimate to the media before we started video recording. Video recording was manually started after the animal completed at least one full stride, and the body was no longer curled. Each animal was recorded for four minutes at the frame rate of 7.5 frames per second. Control animals were tracked on the same day along with animals from test groups. For each genotype, at least 20 animals with equal numbers of males and females were tracked.

### Imaging hardware

The system includes a stereo microscope (SZ61, Olympus America, Center Valley, PA), a digital camera (Fire-i 501b, Unibrain, San Ramon, CA), a motorized stage (H105 ProScan, PRIOR Scientific, Rockland, MA) and a stage controller (ProScan II Controller, PRIOR Scientific, Rockland, MA).

The WormTracker 2.0 software [15] was used to interface with the hardware. The original program supports USB cameras. We modified the program so that it also supports IEEE 1394 cameras. Our modified version can be downloaded from http://wormLoco.org/Mag. The WormTracker controls the motorized stage to continuously center the larva while recording a video and the stage coordinates.

### Image processing

MagRecognizer is the software component that handles image processing. Image processing was performed using ImageJ [35] API library and native codes. First, image frames were extracted from video and binarized. Each binary image was then processed to create a skeleton curve along the midline of the animal. The skeleton curve was divided into 12 segments of equal length, and a total 13 of points were taken from the ends of the segments. The coordinates of the 13 points were then mapped to positions on the agar plate using the stage coordinates. The units were also converted from pixels to millimetres.

## Additional files

Additional file 1: Table S1. Pearson correlation coefficient (PCC) among all parameters. Description of data: Red, PCC>0.7 and PCC<−0.7. Additional file 2: Table S2. Factors affecting locomotive parameter values. Description of data: This table analyzes the same data shown in Figure 4. Data are mean ± standard deviation. *p* values are calculated using student’s *t*-test. Red, *p* < 0.001. Additional file 3: Table S3. Parameter values of circadian gene mutants. Description of data: This table shows normalized parameter values of all mutants shown in Figure 5B. Data are mean ± standard deviation. *n* indicates the number of animals tested. Red, *p* < 0.001 between mutant and control using one-way ANOVA and Scheffe post hoc test. Additional file 4: Table S4. Parameter values of neuronal excitability gene mutants. Description of data: This table shows normalized parameter values of all mutants shown in Figure 6. Data are mean ± standard deviation. *n* indicates the number of animals tested. Red, *p* < 0.001 between mutant and control using one-way ANOVA and Scheffe post hoc test.
