# Supplementary material for: An automated system for quantitative analysis of Drosophila larval locomotion
Source: BMC Dev Biol. 2015 Feb 24;15:11. doi: 10.1186/s12861-015-0062-0 (PMC4345013; doi:10.1186/s12861-015-0062-0)
Supplement: Additional file 1: Table S1. — Pearson correlation coefficient (PCC) among all parameters. Description of data: Red, PCC>0.7 and PCC<−0.7. [file 12861_2015_62_MOESM1_ESM.pdf]

Table S1. Pearson correlation coefficient (PCC) of parameters. Red, PCC>0.7 and PCC<-0.7.

Parameter	Body Length	Body Length Contracted	Body Length Extended	Speed	Time Striding	Speed Striding	Stride Duration	Stride Distance	Contraction Rate	Extension Rate	Stride Count	Run Count	Distance	Time Inside
Body Length	1.00	0.99	0.99	0.39	0.05	0.39	-0.16	0.50	0.35	0.36	0.14	0.00	0.40	-0.29
Body Length Contracted	0.99	1.00	0.97	0.41	0.08	0.43	-0.22	0.49	0.31	0.34	0.20	0.04	0.42	-0.30
Body Length Extended	0.99	0.97	1.00	0.41	0.10	0.39	-0.14	0.55	0.42	0.40	0.14	-0.04	0.41	-0.27
Speed	0.39	0.41	0.41	1.00	0.36	0.94	-0.71	0.83	0.52	0.63	0.71	0.34	0.98	-0.32
Time Striding	0.05	0.08	0.10	0.36	1.00	0.32	-0.37	0.30	0.20	0.33	0.74	-0.06	0.42	-0.22
Speed Striding	0.39	0.43	0.39	0.94	0.32	1.00	-0.80	0.80	0.52	0.63	0.76	0.37	0.96	-0.33
Stride Duration	-0.16	-0.22	-0.14	-0.71	-0.37	-0.80	1.00	-0.40	-0.32	-0.58	-0.83	-0.44	-0.75	0.27
Stride Distance	0.50	0.49	0.55	0.83	0.30	0.80	-0.40	1.00	0.57	0.50	0.42	0.08	0.83	-0.30
Contraction Rate	0.35	0.31	0.42	0.52	0.20	0.52	-0.32	0.57	1.00	0.43	0.31	0.06	0.53	-0.13
Extension Rate	0.36	0.34	0.40	0.63	0.33	0.63	-0.58	0.50	0.43	1.00	0.55	0.21	0.64	-0.12
Stride Count	0.14	0.20	0.14	0.71	0.74	0.76	-0.83	0.42	0.31	0.55	1.00	0.27	0.77	-0.29
Run Count	0.00	0.04	-0.04	0.34	-0.06	0.37	-0.44	0.08	0.06	0.21	0.27	1.00	0.31	-0.05
Distance	0.40	0.42	0.41	0.98	0.42	0.96	-0.75	0.83	0.53	0.64	0.77	0.31	1.00	-0.32
Time Inside	-0.29	-0.30	-0.27	-0.32	-0.22	-0.33	0.27	-0.30	-0.13	-0.12	-0.29	-0.05	-0.32	1.00
