# Supplementary material for: An automated system for quantitative analysis of Drosophila larval locomotion
Source: BMC Dev Biol. 2015 Feb 24;15:11. doi: 10.1186/s12861-015-0062-0 (PMC4345013; doi:10.1186/s12861-015-0062-0)
Supplement: Additional file 2: Table S2. — Factors affecting locomotive parameter values. Description of data: This table analyzes the same data shown in Figure 4. Data are mean ± standard deviation. p values are calculated using student’s t-test. Red, p < 0.001. [file 12861_2015_62_MOESM2_ESM.pdf]

Table S2. Factors affecting locomotive parameters. This table analyzes the same data shown in Figure 4. Data are mean ± standard deviation. *p* values are calculated using student's t-test. Red, $p < 0.001$.

Parameter	Female (n=283)	Male (n=329)	*p*	2nd instar (n=19)	3rd instar (n=31)	*p*
Body Length (mm)	4.41±0.37	4.24±0.30	10^{-9}	1.25±0.27	4.58±0. 31	10^{-33}
Body Length Contracted (mm)	4.19±0.36	4.02±0.29	10^{-9}	1.19±0.26	4.36±0.30	10^{-34}
Body Length Extended (mm)	4.70±0.40	4.53±0.32	10^{-8}	1.30±0.29	4.85±0.33	10^{-34}
Speed (mm/sec)	0.60±0.20	0.63±0.21	0.13	0.097±0.034	0.62±0.12	10^{-22}
Time Striding (%)	75±14	76±12	0.49	30±13	78±14	10^{-14}
Speed Striding (mm/sec)	0.61±0.20	0.63±0.19	0.26	0.11±0.04	0.64±0.12	10^{-23}
Stride Duration (sec)	1.64±0.43	1.58±0.33	0.06	1.95±0.30	1.59±0.27	10^{-4}
Stride Distance (mm)	0.88±0.17	0.89±0.16	0.52	0.19±0.06	0.97±0.10	10^{-34}
Contraction Rate (mm/sec)	0.69±0.15	0.70±0.14	0.50	0.12±0.04	0.71±0.13	10^{-23}
Extension Rate (mm/sec)	0.83±0.19	0.82±0.18	0.69	0.13±0.05	0.65±0.12	10^{-23}
Stride Count (count/minute)	29.5±9.0	30.2±8.1	0.31	9.6±4.9	32.0±10.1	10^{-13}
Run Count (count/minute)	2.17±0.91	2.34±0.95	0.03	1.29±1.01	1.64±0.86	0.22
Distance (mm/minute)	33±11	34±11	0.24	21±8	35±7	10^{-6}
Time Inside (%)	62±23	61±24	0.71	100±0	41±25	10^{-12}

Parameter	Inside (n=625)	Outside (n=573)	*p*
Speed (mm/sec)	0.62±0.21	0.63±0.21	0.78
Time Striding (%)	0.83±0.14	0.70±0.15	10^{-50}
Speed Striding (mm/sec)	0.61±0.19	0.64±0.19	0.06
Stride Duration (sec)	1.63±0.41	1.56±0.34	0.003
Stride Distance (mm)	0.89±0.16	0.90±0.19	0.44
Contraction Rate (mm/sec)	0.69±0.16	0.71±0.17	0.10
Extension Rate (mm/sec)	0.82±0.19	0.85±0.21	0.008
Distance (mm/minute)	33±11	33±15	0.56
