# Supplementary material for: An automated system for quantitative analysis of Drosophila larval locomotion
Source: BMC Dev Biol. 2015 Feb 24;15:11. doi: 10.1186/s12861-015-0062-0 (PMC4345013; doi:10.1186/s12861-015-0062-0)
Supplement: Additional file 3: Table S3. — Parameter values of circadian gene mutants. Description of data: This table shows normalized parameter values of all mutants shown in Figure 5B. Data are mean ± standard deviation. n indicates the number of animals tested. Red, p < 0.001 between mutant and control using one-way ANOVA and Scheffe post hoc test. [file 12861_2015_62_MOESM3_ESM.pdf]

Table S5. Parameter values of circadian gene mutants. This table shows normalized parameter values of all mutants shown in Figure 5B. Data are mean ± standard deviation. n indicates the number of animals tested. Red, $p < 0.001$ between mutant and control using one-way ANOVA and Scheffe post hoc test.

	n	Body Length Contracted	Body Length Extended	Speed	Time Striding	Stride Count	Stride Duration	Stride Distance	Speed Striding	Contraction Rate	Extension Rate	Distance	Time Inside
CS	68	1 ± 0.05	1 ± 0.05	1 ± 0.25	1 ± 0.17	1 ± 0.27	1 ± 0.18	1 ± 0.11	1 ± 0.2	1 ± 0.17	1 ± 0.22	1 ± 0.23	1 ± 0.34
Clk	42	1.01 ± 0.09	1.01 ± 0.09	**0.66 ± 0.26**	**0.68 ± 0.31**	**0.58 ± 0.36**	**1.26 ± 0.32**	**0.79 ± 0.14**	**0.67 ± 0.25**	**0.82 ± 0.17**	**0.74 ± 0.19**	0.66 ± 0.27	1.13 ± 0.5
Per	11	**0.91 ± 0.05**	**0.91 ± 0.06**	0.82 ± 0.12	0.97 ± 0.12	0.97 ± 0.14	0.99 ± 0.1	0.89 ± 0.08	0.88 ± 0.14	1.08 ± 0.21	**0.74 ± 0.16**	0.86 ± 0.14	0.95 ± 0.37
w	83	1 ± 0.05	1 ± 0.06	1 ± 0.19	1 ± 0.29	1 ± 0.34	1 ± 0.15	1 ± 0.12	1 ± 0.18	1 ± 0.18	1 ± 0.15	1 ± 0.2	1 ± 0.24
Pdf	17	**1.12 ± 0.08**	1.1 ± 0.09	0.75 ± 0.32	1.06 ± 0.51	0.88 ± 0.57	**1.29 ± 0.38**	0.88 ± 0.21	**0.74 ± 0.29**	**0.76 ± 0.19**	1.02 ± 0.29	0.74 ± 0.34	1.15 ± 0.28
timeout	31	0.94 ± 0.19	0.94 ± 0.18	0.86 ± 0.24	1.11 ± 0.24	1.05 ± 0.32	1.11 ± 0.27	**0.84 ± 0.12**	**0.8 ± 0.24**	**0.81 ± 0.19**	1.12 ± 0.24	0.85 ± 0.26	0.86 ± 0.47
tim	23	1.05 ± 0.07	1.04 ± 0.07	1.04 ± 0.31	1.2 ± 0.35	1.21 ± 0.47	1.01 ± 0.25	1.01 ± 0.21	1.03 ± 0.27	0.82 ± 0.25	1.04 ± 0.27	1.04 ± 0.33	**0.58 ± 0.26**
