# Supplementary material for: An automated system for quantitative analysis of Drosophila larval locomotion
Source: BMC Dev Biol. 2015 Feb 24;15:11. doi: 10.1186/s12861-015-0062-0 (PMC4345013; doi:10.1186/s12861-015-0062-0)
Supplement: Additional file 4: Table S4. — Parameter values of neuronal excitability gene mutants. Description of data: This table shows normalized parameter values of all mutants shown in Figure 6. Data are mean ± standard deviation. n indicates the number of animals tested. Red, p < 0.001 between mutant and control using one-way ANOVA and Scheffe post hoc test. [file 12861_2015_62_MOESM4_ESM.pdf]

Table S4. Parameter values of neuronal excitability gene mutants. This table shows normalized parameter values of all mutants shown in Figure 6. Data are mean ± standard deviation. n indicates the number of animals tested. Red, $p < 0.001$ between mutant and control using one-way ANOVA and Scheffe post hoc test.

	n	Body Length Contracted	Body Length Extended	Speed	Time Striding	Stride Count	Stride Duration	Stride Distance	Speed Striding	Contraction Rate	Extension Rate	Distance	Time Inside
CS	222	1 ± 0.06	1 ± 0.06	1 ± 0.23	1 ± 0.16	1 ± 0.25	1 ± 0.16	1 ± 0.14	1 ± 0.2	1 ± 0.18	1 ± 0.19	1 ± 0.22	1 ± 0.32
para	20	0.98 ± 0.08	0.99 ± 0.1	0.95 ± 0.22	0.87 ± 0.16	0.84 ± 0.2	1.04 ± 0.17	1.01 ± 0.12	0.98 ± 0.15	1.12 ± 0.24	0.9 ± 0.23	0.95 ± 0.19	1.07 ± 0.44
Hk	43	1 ± 0.05	1 ± 0.06	0.97 ± 0.22	0.87 ± 0.14	0.92 ± 0.17	0.94 ± 0.13	0.91 ± 0.14	0.96 ± 0.23	0.97 ± 0.25	0.89 ± 0.18	0.96 ± 0.22	0.88 ± 0.36
eag	51	1 ± 0.07	1 ± 0.08	0.89 ± 0.22	**0.74 ± 0.27**	**0.69 ± 0.28**	1.06 ± 0.22	**0.86 ± 0.15**	**0.83 ± 0.21**	0.9 ± 0.17	**0.75 ± 0.18**	0.88 ± 0.23	1.14 ± 0.42
Sh	84	0.98 ± 0.06	**0.97 ± 0.07**	**0.65 ± 0.23**	**0.73 ± 0.32**	**0.57 ± 0.3**	**1.36 ± 0.34**	**0.84 ± 0.17**	**0.66 ± 0.22**	**0.77 ± 0.19**	**0.71 ± 0.19**	**0.65 ± 0.22**	**1.36 ± 0.43**
slo	71	**0.91 ± 0.05**	**0.9 ± 0.05**	0.88 ± 0.23	0.86 ± 0.22	0.87 ± 0.27	0.99 ± 0.16	**0.86 ± 0.12**	0.9 ± 0.22	**0.77 ± 0.15**	**0.82 ± 0.14**	0.86 ± 0.22	1.22 ± 0.43
dnc	21	1.02 ± 0.04	**1.02 ± 0.04**	1.25 ± 0.27	1.08 ± 0.13	1.17 ± 0.27	0.88 ± 0.18	**1.16 ± 0.09**	**1.27 ± 0.28**	1.09 ± 0.23	0.83 ± 0.12	**1.29 ± 0.3**	0.75 ± 0.32
